# Supplementary material for: Single-Cell Transcriptional and Epigenetic Profiles of Male Breast Cancer Nominate Salient Cancer-Specific Enhancers
Source: Int J Mol Sci. 2023 Aug 22;24(17):13053. doi: 10.3390/ijms241713053 (PMC10487538; doi:10.3390/ijms241713053)
Supplement: Supplementary file 1 [file ijms-24-13053-s001.zip › Supplemental Figures MaleBC Paper.pdf]

Single-Cell Transcriptional and Epigenetic Profiles of Male Breast Cancer Nominate Salient Cancer-Specific Enhancers

Hyunsoo Kim[1,6], Kamila Wisniewska[1,6], Matthew J. Regner[1,2], Aatish Thennavan[1,3], Philip M. Spanheimer[1,4] and Hector L. Franco[1,2,5*]

Supplementary Information

1. Supplementary Figure S1. Quality metrics for scRNA-seq and scATAC-seq.

2. Supplementary Figure S2. CNV Thresholds for scRNA-seq Cells and QC Thresholds for scATAC-seq Cells

3. Supplementary Figure S3. Peak-to-Gene Link Analysis Identifies Potential *LAMB3* Super-Enhancer and Confirms Previously Annotated *CD47* Super-Enhancer

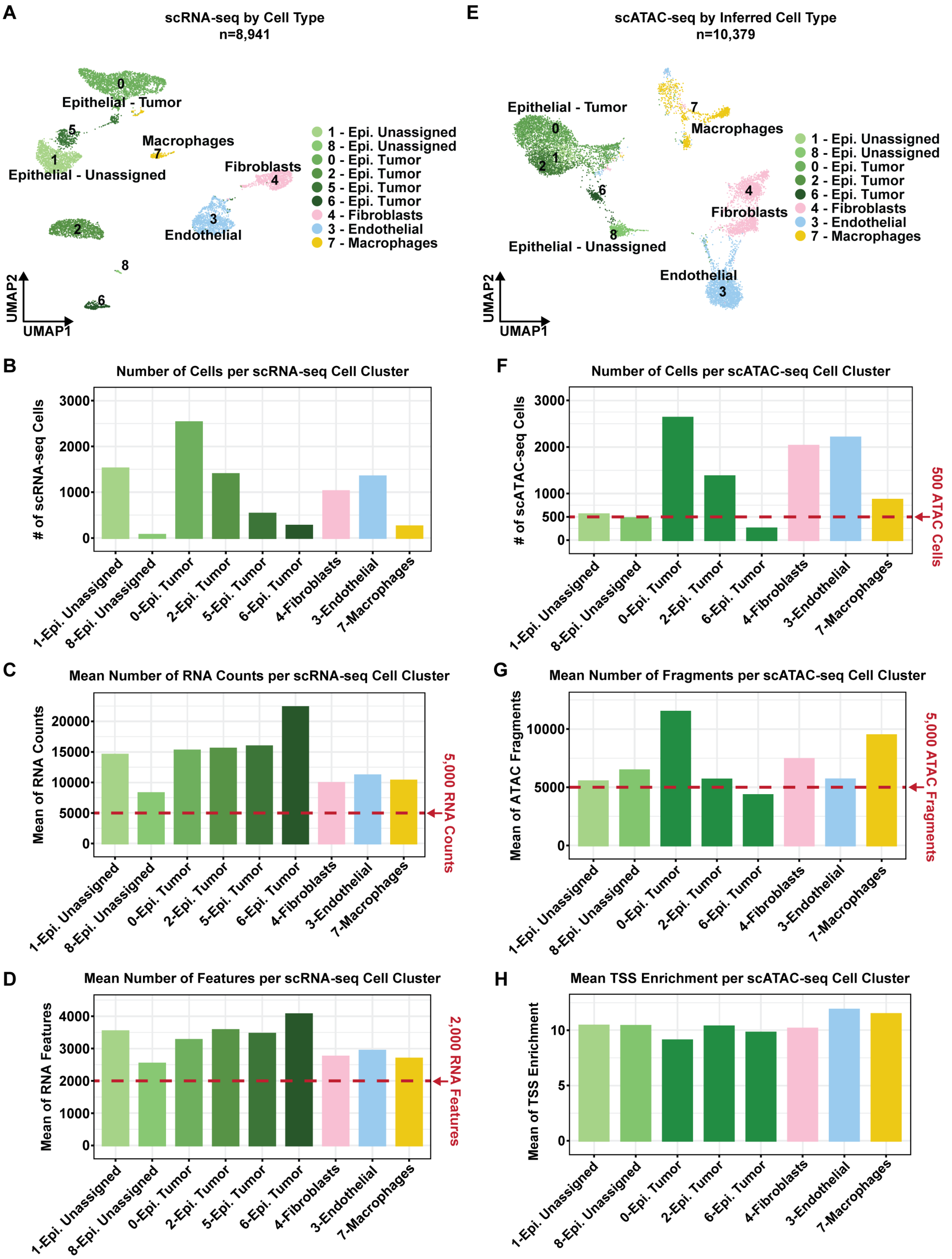
A
scRNA-seq by Cell Type
n=8,941
Epithelial - Tumor
Macrophages
Epithelial - Unassigned
Fibroblasts
Endothelial
1 - Epi. Unassigned
8 - Epi. Unassigned
0 - Epi. Tumor
2 - Epi. Tumor
5 - Epi. Tumor
6 - Epi. Tumor
4 - Fibroblasts
3 - Endothelial
7 - Macrophages
UMAP2
UMAP1
E
scATAC-seq by Inferred Cell Type
n=10,379
Epithelial - Tumor
Macrophages
Fibroblasts
Epithelial - Unassigned
Endothelial
1 - Epi. Unassigned
8 - Epi. Unassigned
0 - Epi. Tumor
2 - Epi. Tumor
6 - Epi. Tumor
4 - Fibroblasts
3 - Endothelial
7 - Macrophages
B
Number of Cells per scRNA-seq Cell Cluster
# of scRNA-seq Cells
F
Number of Cells per scATAC-seq Cell Cluster
# of scATAC-seq Cells
500 ATAC Cells
C
Mean Number of RNA Counts per scRNA-seq Cell Cluster
Mean of RNA Counts
5,000 RNA Counts
G
Mean Number of Fragments per scATAC-seq Cell Cluster
Mean of ATAC Fragments
5,000 ATAC Fragments
D
Mean Number of Features per scRNA-seq Cell Cluster
Mean of RNA Features
2,000 RNA Features
H
Mean TSS Enrichment per scATAC-seq Cell Cluster
Mean of TSS Enrichment
1-Epi. Unassigned
8-Epi. Unassigned
0-Epi. Tumor
2-Epi. Tumor
5-Epi. Tumor
6-Epi. Tumor
4-Fibroblasts
3-Endothelial
7-Macrophages

Supplementary Figure S1. Quality metrics for scRNA-seq and scATAC-seq.

A. scRNA-seq UMAP plot colored by cell-type, as in Fig. 1B, and numbered by cell-type cluster.
B. Bar chart showing the number of scRNA-seq cells in each cell-type cluster.
C. Bar chart showing the mean number of RNA counts per cell-type cluster. Red line denotes the cutoff threshold of 5,000 RNA counts.
D. Bar chart showing the mean number of RNA features per cell-type cluster. Red line denotes cutoff threshold of 2,000 RNA features.
E. scATAC-seq UMAP plot colored by cell type and numbered by cell-type cluster prior to filtering out low quality cells. Three cell-type clusters 1-Epi. Unassigned, 8-Epi. Unassigned, and 6-Epi. Tumor were filtered out in the UMAP in Fig. 1B.
F. Bar chart showing the number of scATAC-seq cells in each cell-type cluster. The red line denotes the cutoff threshold of 500 ATAC cells.
G. Bar chart showing the mean number of fragments per scATAC-seq cell-type cluster. Red line denotes the cutoff threshold of 5,000 ATAC fragments.
H. Bar chart showing the mean TSS enrichment per scATAC-seq cell-type cluster.

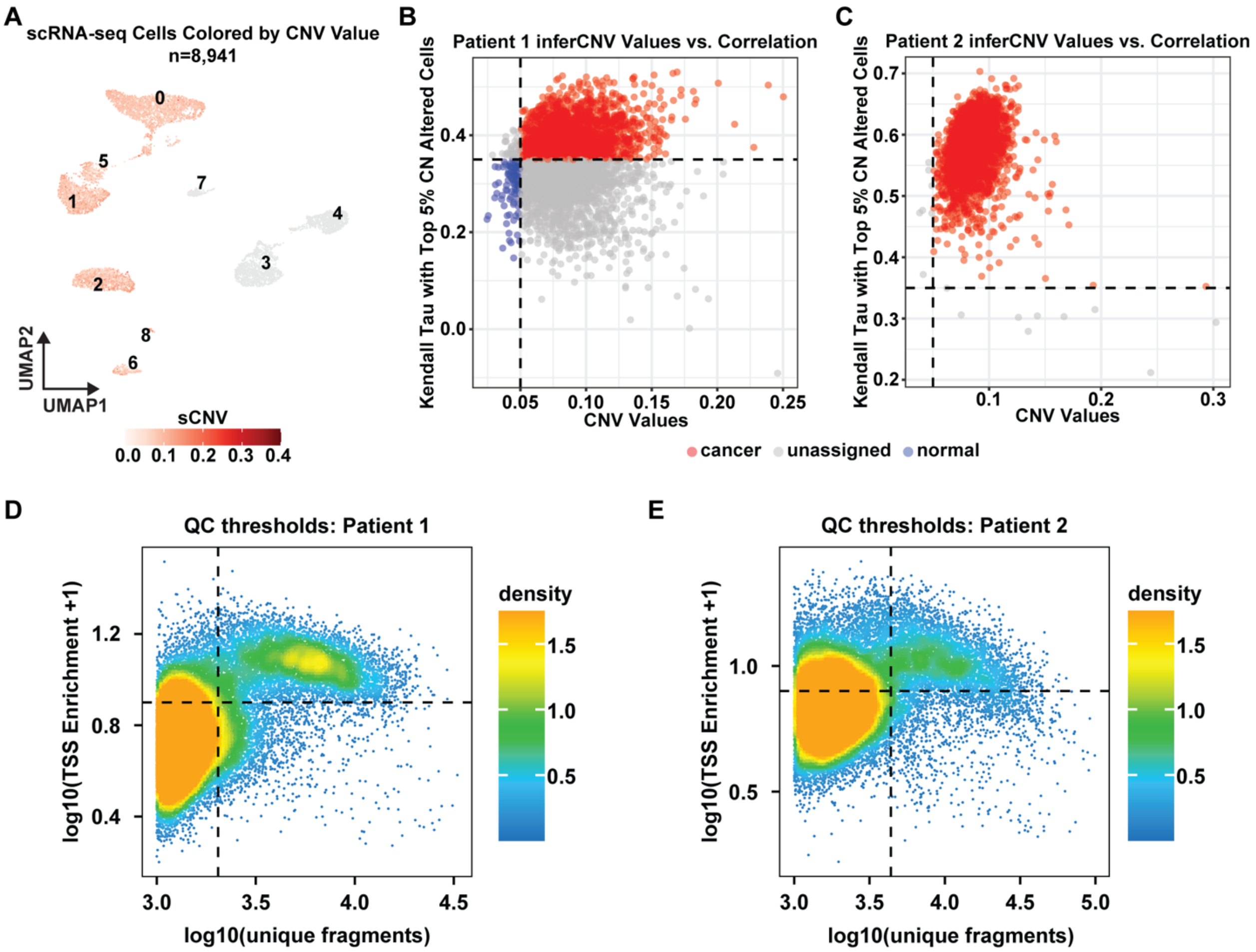

Supplementary Figure S2. CNV Thresholds for scRNA-seq Cells and QC Thresholds for scATAC-seq Cells

A. scRNA-seq UMAP plot colored by CNV value.

B. Scatterplot of Patient 1 inferCNV values vs. correlation. Correlation is measured as Kendall Tau with top 5% CN altered cells. Cells in red are considered cancer cells, cells in grey are considered unassigned cells, and cells in blue are considered normal cells.

C. Scatterplot of Patient 2 inferCNV values vs. correlation. Correlation is measured as Kendall Tau with top 5% CN altered cells. Cells in red are considered cancer cells, cells in grey are considered unassigned cells, and cells in blue are considered normal cells.

D. Scatterplot of barcode QC for Patient 1 scATAC-seq based on thresholds of TSS enrichment and log10(unique fragments).

E. Scatterplot of barcode QC for Patient 2 scATAC-seq based on thresholds of TSS enrichment and log10(unique fragments).

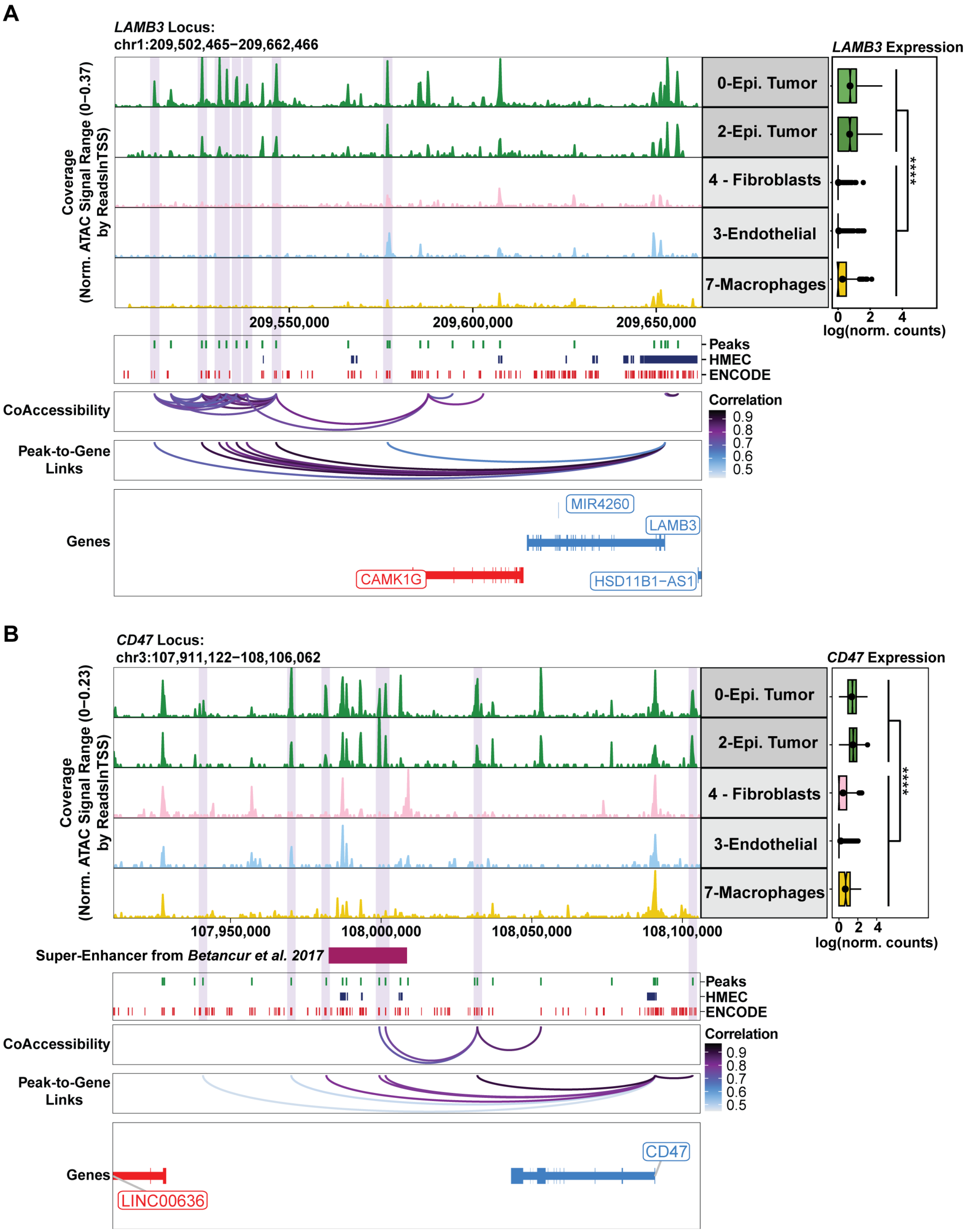
A
LAMB3 Locus:
chr1:209,502,465−209,662,466
Coverage
(Norm. ATAC Signal Range (0−0.37)
by ReadsInTSS)
0-Epi. Tumor
2-Epi. Tumor
4 - Fibroblasts
3-Endothelial
7-Macrophages
LAMB3 Expression
****
0 2 4
log(norm. counts)
209,550,000
209,600,000
209,650,000
Peaks
HMEC
ENCODE
CoAccessibility
Correlation
0.9
0.8
0.7
0.6
0.5
Peak-to-Gene
Links
Genes
MIR4260
LAMB3
CAMK1G
HSD11B1−AS1
B
CD47 Locus:
chr3:107,911,122−108,106,062
Coverage
(Norm. ATAC Signal Range (0−0.23)
by ReadsInTSS)
0-Epi. Tumor
2-Epi. Tumor
4 - Fibroblasts
3-Endothelial
7-Macrophages
CD47 Expression
****
0 2 4
log(norm. counts)
107,950,000
108,000,000
108,050,000
108,100,000
Super-Enhancer from Betancur et al. 2017
Peaks
HMEC
ENCODE
CoAccessibility
Correlation
0.9
0.8
0.7
0.6
0.5
Peak-to-Gene
Links
Genes
CD47
LINC00636

Supplementary Figure S3. Peak-to-Gene Link Analysis Identifies Potential *LAMB3* Super-Enhancer and Confirms Previously Annotated *CD47* Super-Enhancer

A. Browser track view of chromatin accessibility at the *LAMB3* locus across epithelial cancer cells (Epi. Tumor) and all other cell-type clusters. Predicted enhancers of *LAMB3* are highlighted in light purple. The peak track below the browser track denotes all scATAC-seq peaks from this study (Peaks), regulatory elements found in human mammary epithelial cells (HMEC), and all ENCODE cis-regulatory elements. The peak-to-gene correlation loops show the correlation between *LAMB3* and the peaks linked to this gene. Gene expression of *LAMB3* in matched scRNA-seq cells is depicted to the right of the browser track. **** denote *p*-value < 2.22e-16 (Wilcoxon rank-sum test).

B. Browser track view of chromatin accessibility at the *CD47* locus across epithelial cancer cells (Epi. Tumor) and all other cell-type clusters. Predicted enhancers of *CD47* are highlighted in light purple. The *CD47* super-enhancer from Betancur *et al*.[59] is denoted in magenta. The peak track below the browser track denotes all scATAC-seq peaks from this study (Peaks), regulatory elements found in human mammary epithelial cells (HMEC), and all ENCODE cis-regulatory elements. The peak-to-gene correlation loops show the correlation between *CD47,* and the peaks linked to this gene. Gene expression of *CD47* in matched scRNA-seq cells is depicted to the right of the browser track. **** denote *p*-value < 2.22e-16 (Wilcoxon rank-sum test).
